# The impact of geographic inequality in federal research funding: A comparative longitudinal study of research and scholarly outputs in EPSCoR versus non-EPSCoR states

**DOI:** 10.1371/journal.pone.0286991

**Published:** 2023-06-16

**Authors:** Ehsan Mohammadi, Anthony J. Olejniczak, George E. Walker, Prakash Nagarkatti

**Affiliations:** 1 School of Information Science, University of South Carolina, Columbia, SC, United States of America; 2 Academic Analytics Research Center, Columbus, OH, United States of America; 3 University of South Carolina, Columbia, SC, United States of America; Max Planck Institute for Solid State Research, GERMANY

## Abstract

Some states in the U.S. have traditionally received less federal research funding than other states. The National Science Foundation (NSF) created a program in 1979, called the Experimental Program to Stimulate Competitive Research (EPSCoR) to enhance the research competitiveness in such states. While the geographic disparity in federal research funding is well known, the overall impact of federal funding on the research performance of EPSCoR and non-EPSCoR has not been previously studied. In the current study, we compared the combined research productivity of Ph.D. granting institutions in EPSCoR versus the non-EPSCoR states to better understand the scientific impact of federal investments in sponsored research across all states. The research outputs we measured included journal articles, books, conference papers, patents, and citation count in academic literature. Unsurprisingly, results indicated that the non-EPSCoR states received significantly more federal research funding than their EPSCoR counterparts, which correlated with a higher number of faculty members in the non-EPSCoR versus EPSCoR states. Also, in the overall research productivity expressed on a per capita, the non-EPSCoR states fared better than EPSCoR states. However, when the research output was measured based on per $1M investment of federal research funding, EPSCoR states performed significantly better than the non-EPSCoR states in many research productivity indicators, with the notable exception of patents. Together, this study found preliminary evidence that EPSCoR states achieved a high degree of research productivity despite receiving significantly fewer federal research dollars. The limitations and next steps of this study are also discussed.

## 1. Introduction

When the National Science Foundation (NSF) was created, the Congress encouraged this federal agency to develop strategies “to strengthen research and education in science and engineering throughout the United States and to avoid undue concentration of such research and education.” However, over the years, with the increase in academic research support from NSF and other federal agencies, it was noted that there was a significant disparity in the levels of federal research funding received by various states, thereby creating geographic disparity.

Because federal support for research drives innovation and economic prosperity, the disparity in Federal funding creates geographic economic inequality which has been a concern to the leadership at the EPSCoR states as well as the United States Congress, as evidenced by recent and ongoing debates [1]. In 1978, the National Science Board, which directs NSF, endorsed creating a new program called the Experimental Program to Stimulate Competitive Research (EPSCoR) to help overcome the disparity in research support to some states that were less competitive in securing Federal support. Thus, the goal of the NSF’s EPSCoR program, as stated in the NSF mission, was to “enhance the research competitiveness of targeted jurisdictions (states, territories, and a commonwealth) by strengthening STEM capacity through strategic investments involving talent development to regional infrastructure”.

In the years to follow, other Federal agencies, including the Department of Energy (DoE), the Department of Defense (DoD), the National Aeronautics and Space Administration (NASA), the United States Department of Agriculture (USDA), and the like, started similar EPSCoR programs to address the concern over the uneven distribution of federal research dollars among the states. In 1993, the National Institutes of Health (NIH) started a new program with similar goals called the Institutional Development Award (IDeA).

When NSF started the EPSCoR program in 1979, it supported seven states, each totaling about $125,000. These states included Arkansas, Maine, Montana, South Carolina, and West Virginia. However, through bipartisan congressional support, the EPSCoR/IDeA funding has grown significantly. In 2022, the NSF EPSCoR program had grown to $215 million, and NIH allocated $410 million to the IDeA program. This combined with other Federal agencies resulted in a total of $761 million in research funding allocation to the EPSCoR/IDeA jurisdictions. Last year, the United States Senate approved legislation called U.S. Innovation and Competition Act (USICA) that would dedicate a significant increase in the overall budget from NSF and DoE to the EPSCoR states. USICA required 20% of NSF’s budget and 20% of the $7.5 billion budget of the DOE science office to be allocated to the EPSCoR states [1]. The House’s version called the America COMPETES Act of 2022, in contrast, did not specifically allocate direct resources to the EPSCoR states like the USCIA but recommended the creation of programs to build research capacity at universities that are not the top tier research institutions, regardless of where they are located. Currently, the EPSCoR programs receive ∼3% of the NSF budget and the EPSCoR jurisdictions receive ~13% of the NSF budget. Thus, the bill to increase the allocation to EPSCoR states to 20% triggered significant levels of debate on whether such dramatic increases were necessary and whether the EPSCoR states had the capacity to make the best use of such funds. Also, many university leaders from non-EPSCoR states felt that a 20% allocation to EPSCoR would unfairly prevent them from competing nationally for additional research resources [2].

To overcome this criticism, there was a compromise, and the CHIP and Science act was ultimately passed in August 2022. This legislation requires NSF to increase the research funding to EPSCoR states from the current level funding of 13% to 20% by 2029. Also, a similar 20% target is required to be accomplished in all NSF scholarships and training programs. Additionally, the Department of Energy is expected to increase funding for EPSCoR programs and allocate at least 10% of the Office of Science academic research funding to the EPSCoR states [1]. The substantial increase in NSF and DOE funding to the EPSCoR states, as proposed in the CHIPS and Science act, still raises questions on the current research productivity of EPSCoR states and how well-prepared they are for making the best use of such increased research funding to grow their research capacity. Under the America COMPETES Reauthorization Act of 2010, the Congress asked the National Academy of Sciences to measure the effectiveness of the EPSCoR to achieve the mission of building the research infrastructure and capacity in EPSCoR jurisdictions to make them more competitive in research. In 2013, the National Academy of Sciences published a report [3] in which they tried to evaluate the effectiveness of EPSCoR. The committee reported that they were not able to effectively assess EPSCoR with the necessary objectivity because EPSCoR had evolved and broadened its scope over time. Also, the committee reported that the data on operations and outcomes were not readily available to draw conclusions on the effectiveness of the program.

However, the committee also noted that over the years, the EPSCoR states had performed well and enhanced their research capacity. Because EPSCoR funding represents only a small slice of the overall federal research funding that these states receive, the precise impact of EPSCoR was difficult to ascertain. In 2017, after 40 years and a record of significant impact, EPSCoR officially changed from ‘Experimental’ to ‘Established’ Program to Stimulate Competitive Research.

The significant geographic disparity in Federal R&D funding to the states as described above suggests that states that are non-EPSCoR/IDeA, in general, should fare significantly better than EPSCoR/IDeA states in the quality and quantity of research. However, such comparisons have previously not been made systematically. Also, as Congress authorizes to invest more federal dollars into research capacity building in EPSCoR states to promote geographic diversity, understanding the current performance of EPSCoR states in research productivity and impact becomes crucial.

In the current study, we, therefore examined various research productivity indicators such as the number of journal and book publications, number of conference presentations, patents, and the number of citations in the past ∼10 years (2010–2020) among Ph.D. granting universities located in EPSCoR vs non-EPSCoR states. Additionally, because non-EPSCoR states may have overall more faculty researchers than EPSCoR states, the data that we generated were also expressed on a per capita basis. Lastly, we also analyzed and compared the research productivity of the EPSCoR vs non-ESPCoR states for every million dollars of federal research investment.

## 2. Materials and methods

### 2.1. Faculty lists

We mined the Academic Analytics, LLC (AcA) commercial database for the departmental affiliation(s), and year of terminal degree of tenured and tenure-track scholars (Assistant Professor, Associate Professor, and Professor titles) employed by 392 Ph.D.-granting universities in the United States. AcA data have been demonstrated suitable for academic scholarship in several studies [4–7]. AcA faculty rosters are verified and updated annually by manual collection from publicly available resources, supplemented by verification and submission of faculty lists from some institutions. Each academic department is manually assigned to one of 170 subject classifications based on the National Center for Education Statistics (NCES) Classification of Instructional Programs (CIP) code classifications [8]. A list of departments and institutions included in this study, as well as their discipline classifications, are publicly available via OSF (https://osf.io/ur7ga/). Faculty lists were mined for each of 10 consecutive years, beginning with the academic year Fall 2010 –Spring 2011 and ending with the Fall 2020 –Spring 2021 academic year. Institutions were labeled as “EPSCoR” and “non-EPSCoR” based on the jurisdiction/state in which they are located, according to the 2022 eligibility criteria shown at the NSF EPSCoR website (https://beta.nsf.gov/funding/initiatives/epscor/epscor-criteria-eligibility). The AcA database lacks information about Ph.D. granting universities in US territories (both Puerto Rico and the US Virgin Islands are home to Ph.D. granting institutions). We also omitted universities within the District of Columbia, as this jurisdiction is not within the EPSCoR program. Our sample included universities in 25 EPSCoR states and 25 non- EPSCoR states (Table 1).

**Table 1:** The EPSCoR eligibility of each US state and the number of Ph.D. granting universities in that state are catalogued by academic analytics.

State	EPSCoR Status	# Universities	State	EPSCoR Status	# Universities
Alabama	EPSCoR	7	Montana	EPSCoR	2
Alaska	EPSCoR	1	Nebraska	EPSCoR	4
Arizona		3	Nevada	EPSCoR	2
Arkansas	EPSCoR	4	New Hampshire	EPSCoR	3
California		32	New Jersey		12
Colorado		7	New Mexico	EPSCoR	3
Connecticut		4	New York		36
Delaware	EPSCoR	1	North Carolina		10
Florida		14	North Dakota	EPSCoR	2
Georgia		9	Ohio		13
Hawaii	EPSCoR	1	Oklahoma	EPSCoR	3
Idaho	EPSCoR	3	Oregon		4
Illinois		17	Pennsylvania		17
Indiana		6	Rhode Island	EPSCoR	3
Iowa	EPSCoR	3	South Carolina	EPSCoR	3
Kansas	EPSCoR	4	South Dakota	EPSCoR	3
Kentucky	EPSCoR	4	Tennessee		11
Louisiana	EPSCoR	10	Texas		42
Maine	EPSCoR	2	Utah		3
Maryland		8	Vermont	EPSCoR	1
Massachusetts		22	Virginia		10
Michigan		11	Washington		4
Minnesota		2	West Virginia	EPSCoR	2
Mississippi	EPSCoR	5	Wisconsin		5
Missouri		7	Wyoming	EPSCoR	1

### 2.2. Scholarly works

AcA matches scholarly publications, grants, and patents to their authors using a semi-automated matching process. The likelihood of a scholarly work being matched to an author was calculated by, e.g., similarity in names, co-authorship history, and publication title history, and the match and likelihood scores were then evaluated for accuracy. All peer-reviewed journal articles and conference proceedings indexed in CrossRef (https://www.crossref.org/) were imported into a data warehouse and matched to their author(s). Academic book publications from 5,774 publishers indexed by Baker & Taylor (https://www.baker-taylor.com/) were used. Patents were obtained from the US Patent and Trademark Office database (https://www.uspto.gov/patents), and the research grants (both the number of grants and the annualized dollar amount) were obtained from US federal government databases. Federal research grants in the AcA database represent competitive research funding; other forms of funding are not included (*e*.*g*., contract funding, some pass-through grants), so the totals reported here are lower than the total dollars reported, *e*.*g*., in the NSF HERD survey (https://www.nsf.gov/statistics/herd/). Lists of all research elements (journal titles, book publishers, granting agencies, etc.) are available via OSF (https://osf.io/ur7ga/). For each scholarly output type, records from each calendar year were matched to the corresponding year’s faculty rosters. For example, journal articles published in 2011 were matched to scholars in the Fall 2010-Spring 2011 faculty roster. In this way, the institution where the publication, grant, or patent was authored, was credited with the work, even if the scholar moved to a new institution, retired, or otherwise left the study sample during subsequent years. Scholarly works were counted equally for every author; an article with authors in EPSCoR states and non-EPSCoR states was counted in the article total for both groups.

### 2.3. Analysis–EPSCoR and non-EPSCoR

Plots were created showing the change in several variables between 2011 and 2020 among research universities within EPSCoR and non-EPSCoR states. Both raw data (counts, unscaled) and scaled data (*e*.*g*., journal articles per $1 million in grant dollars) were calculated. The following metrics were analyzed:

1. Total federal research grant dollars (unscaled)
2. Faculty count (unscaled)
3. Annualized federal research grant dollars per faculty member
4. Journal articles per faculty member
5. Books per faculty member
6. Conference proceedings per faculty member
7. Citations per faculty member
8. Citations per journal article
9. USPTO Patents per faculty member
10. Journal articles per million federal research grant dollars
11. Books per faculty member
12. Conference proceedings per million federal research grant dollars
13. Citations per million federal research grant dollars
14. Patents per million federal research grant dollars

For each metric, we compared EPSCoR and non-EPSCoR values for all years combined via the Mann-Whitney U test [9]. We opted for this non-parametric alternative to a two-sample t-test because our datasets were not normally distributed. Tests were run in R [10] for each metric describing scholarly works. In addition to the totals and per capita plots described above for EPSCoR and non-EPSCoR faculty members, we also compared state-level metrics for EPSCoR and non-EPSCoR states for each data year individually, as well as annual box plots depicting the median and interquartile range for EPSCoR and non-EPSCoR states. The results of these tests and the boxplots are available via https://osf.io/ur7ga/.

## 3. Results

### 3.1. Federal funding received by EPSCoR vs non-EPSCoR

**Fig 1:**
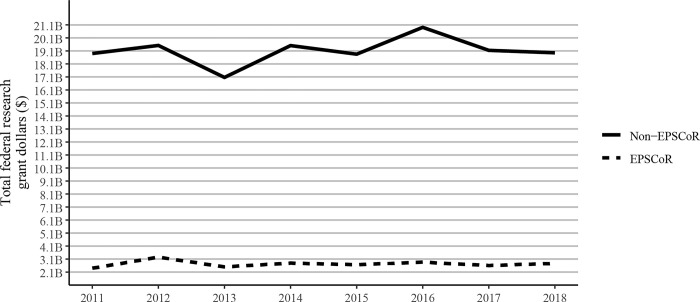
Total federal grant dollars received by investigators at Ph.D. granting universities in EPSCoR and non-EPSCoR states between 2011 and 2020. EPSCoR mean = $2.7B ±$0.78B; non-EPSCoR mean = $19.3B ±$0.78B; p < 0.01.

We used the most recent (2022) NSF criteria to identify EPSCoR and non-EPSCoR states and then generated data on the total federal grant dollars received. As expected, the data shown in Fig 1 demonstrated that the non-EPSCoR states in years 2011 to 2018, received much higher levels of research funding compared to the EPSCoR states.

### 3.2. Total number of faculty in EPSCoR vs non-EPSCoR states

**Fig 2:**
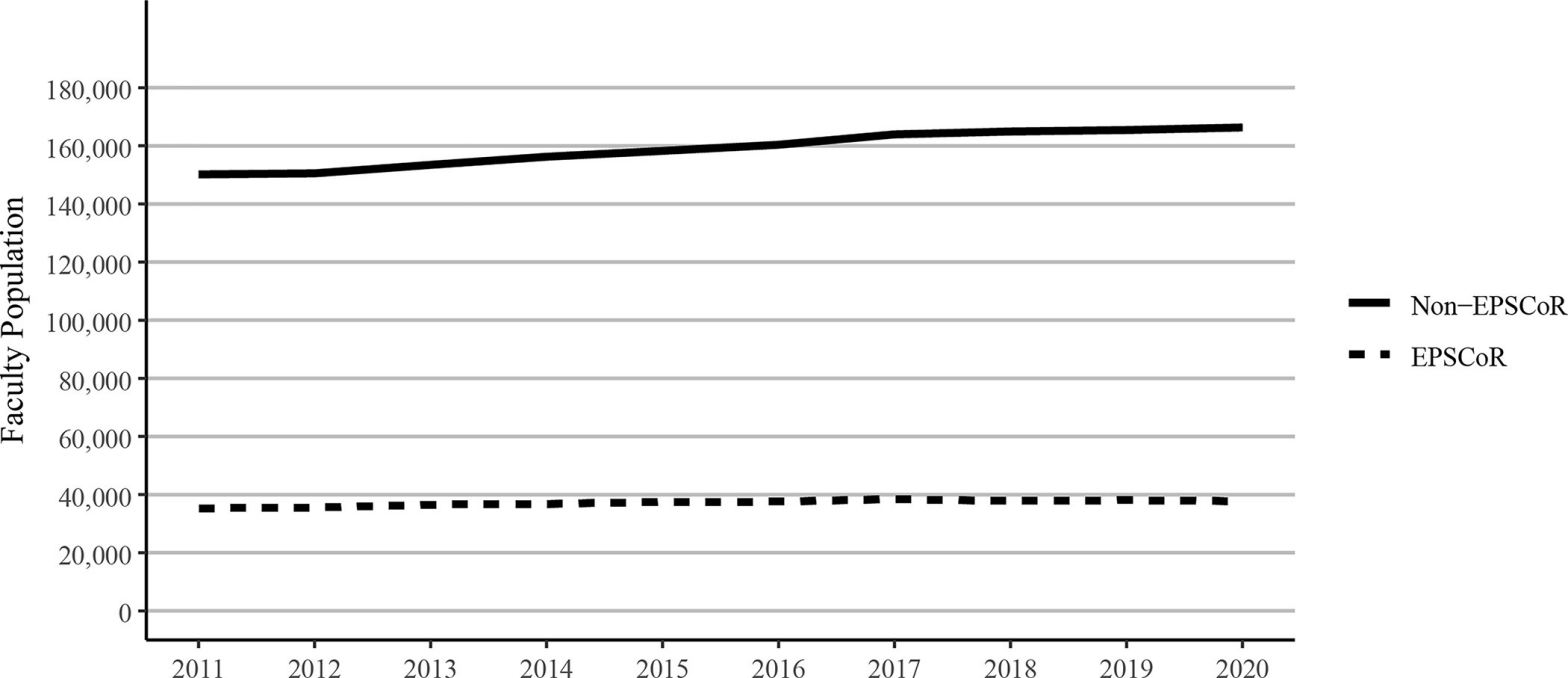
Total faculty numbers at Ph.D. granting universities in EPSCoR and non-EPSCoR states. EPSCoR mean = 36,567 ±345.2B; non-EPSCoR mean = 162,541 ±1,987.0; p<0.01.

Because the total federal research dollars received by any state depends on the number of investigators, we next analyzed the faculty population in Ph.D. granting universities in EPSCoR vs non-EPSCoR states. The population of faculty members at research universities in the EPSCoR states was found to be much lower when compared to the non-EPSCoR states (Fig 2). Moreover, the total number of faculty within EPSCoR states grew at a slower rate than the number of faculty in non-EPSCoR states. The population of faculty members at research universities in non-EPSCoR states increased by 10.8% between 2011 and 2020 (from 153,165 to 166,314 scholars), while the faculty population within EPSCoR states increased by 6.8% (from 35,300 to 37,712 scholars).

### 3.3. Research grants per person

**Fig 3:**
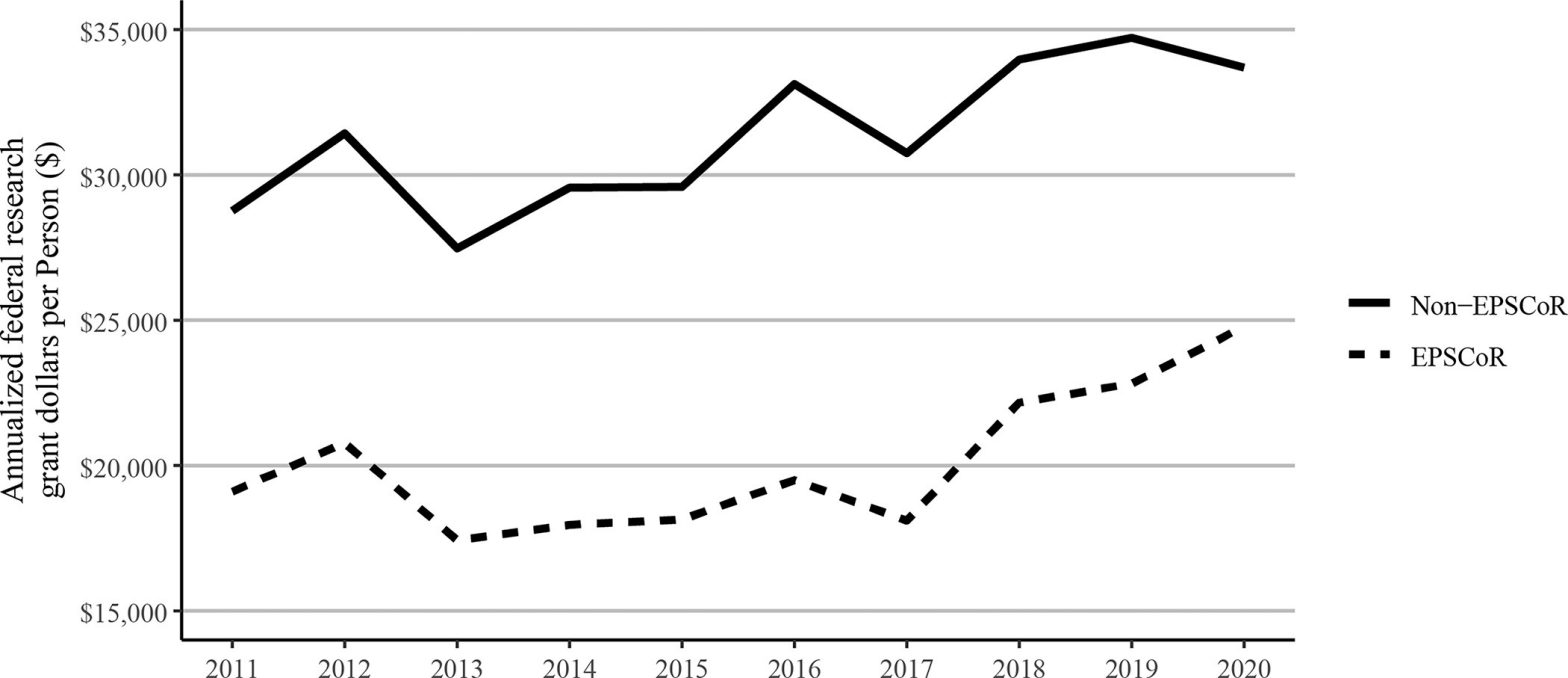
Annualized federal grant dollars per faculty member received at Ph.D. granting universities in EPSCoR and non-EPSCoR states between 2011 and 2020. EPSCoR mean = $20,112 ±$776.2B; non-EPSCoR mean = $30,947 ±$774.5; p < 0.01.

Because there were substantial differences in the total number of faculty in EPSCoR vs non-EPSCoR states, we analyzed and expressed the data on a per capita basis. Annualized federal research grant dollars received by faculty members in EPSCoR states were found to be lower than that received by their non-EPSCoR counterparts (Fig 3). Overall, there was an increase in the annualized grant dollars received per faculty member both in EPSCoR and non-EPSCoR states. On average, EPSCoR states received only 12.7% of the annualized dollars won by non-EPSCoR states in 2011; this gap narrowed slightly by 2018, with EPSCoR state grant dollar totals being 14.6% of the total won by non-EPSCoR states (Fig 1). Federal grant dollars per faculty increased at a greater rate among EPSCoR institutions (29.8%) than non-EPSCoR institutions (17.1%) during the study period (Fig 3), consistent with a narrowing gap in total federal grant dollars and a more slowly growing population of faculty members in EPSCoR states.

### 3.4. Publications per faculty member

**Fig 4:**
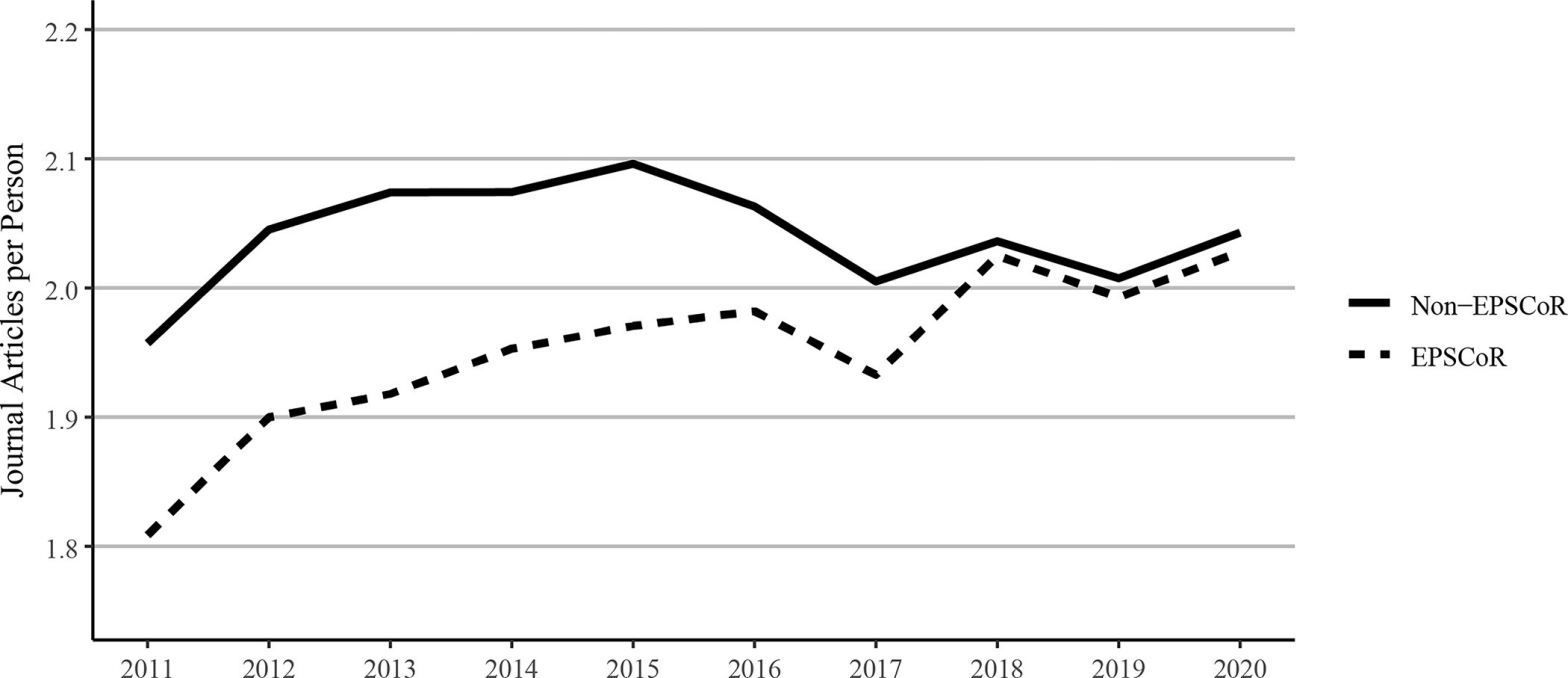
Journal articles per faculty member published between 2011 and 2020 at Ph.D. granting universities in EPSCoR and non-EPSCoR states. EPSCoR mean = 1.95 ±0.020; non-EPSCoR mean = 2.01 ±0.012; p < 0.05.

The number of journal articles published per faculty member in EPSCoR states was significantly lower than their counterparts from non-EPSCoR states in 2011. However, from 2018–2020, this gap narrowed such that there was virtually no difference between the EPSCoR vs non-EPSCoR states. Also, between 2011 and 2020, journal articles per faculty member increased at both EPSCoR and non-EPSCoR institutions (Fig 4) from fewer than 2.0 articles per person per year to greater than 2.0 articles per person per year. This trend is consistent with previous studies demonstrating overall growth in the number of journal articles published annually [11–13].

**Fig 5:**
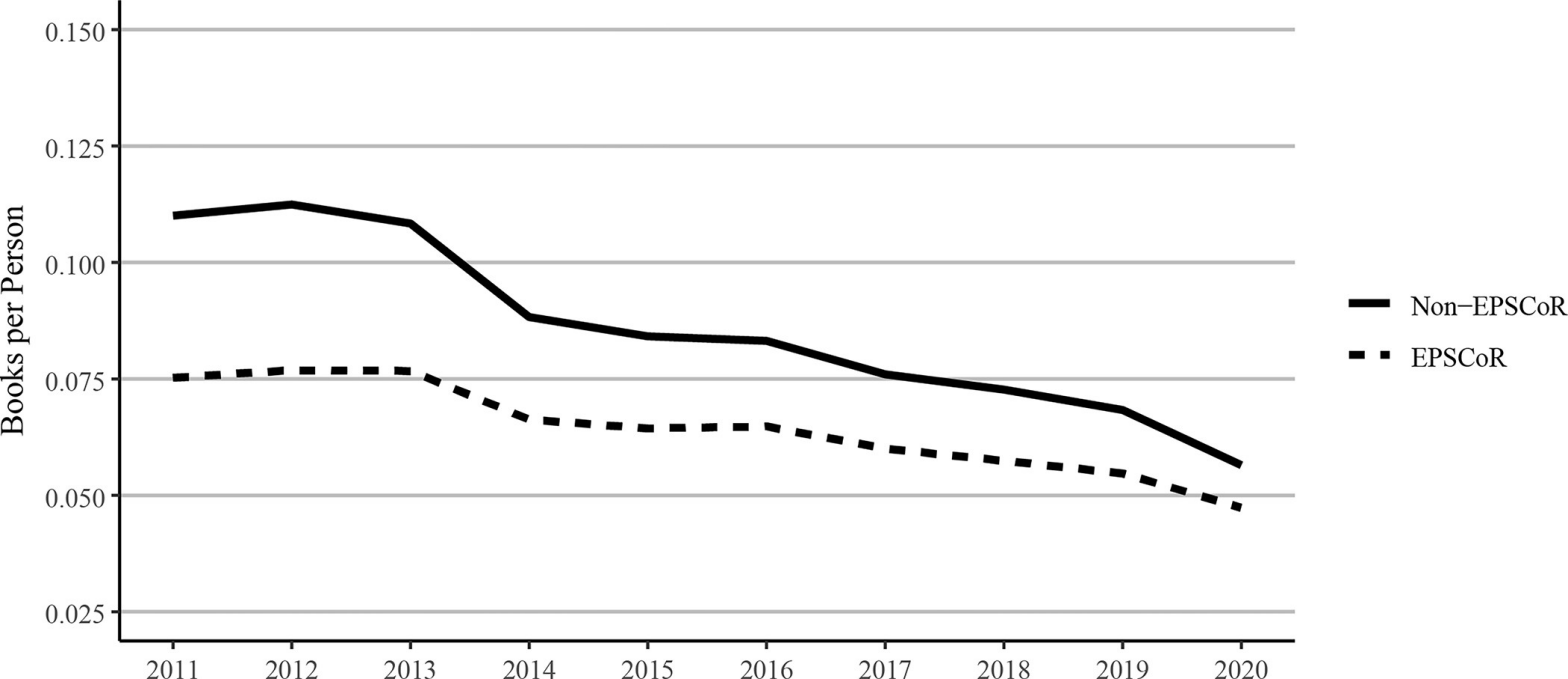
Books per faculty member published between 2011 and 2020 at Ph.D. granting universities in EPSCoR and non-EPSCoR states. EPSCoR mean = 0.065 ±0.0031; non-EPSCoR mean = 0.086 ±0.0060; p < 0.01.

Similar to journal articles, the publication of books per faculty member in EPSCoR states was less than that seen in non- EPSCoR states in 2020. However, this gap narrowed over the study period. Books per person decreased between 2011 and 2020 in both EPSCoR and non-EPSCoR states (Fig 5). Decreasing academic book publications in the social sciences has been documented elsewhere [14], but the breadth of declining books authored across disciplines and universities we document here has not been reported previously, to our knowledge. In 2011, non-EPSCoR state authors published 46.3% more books per person per year on average; in 2020, the gap was only 19.3%.

**Fig 6:**
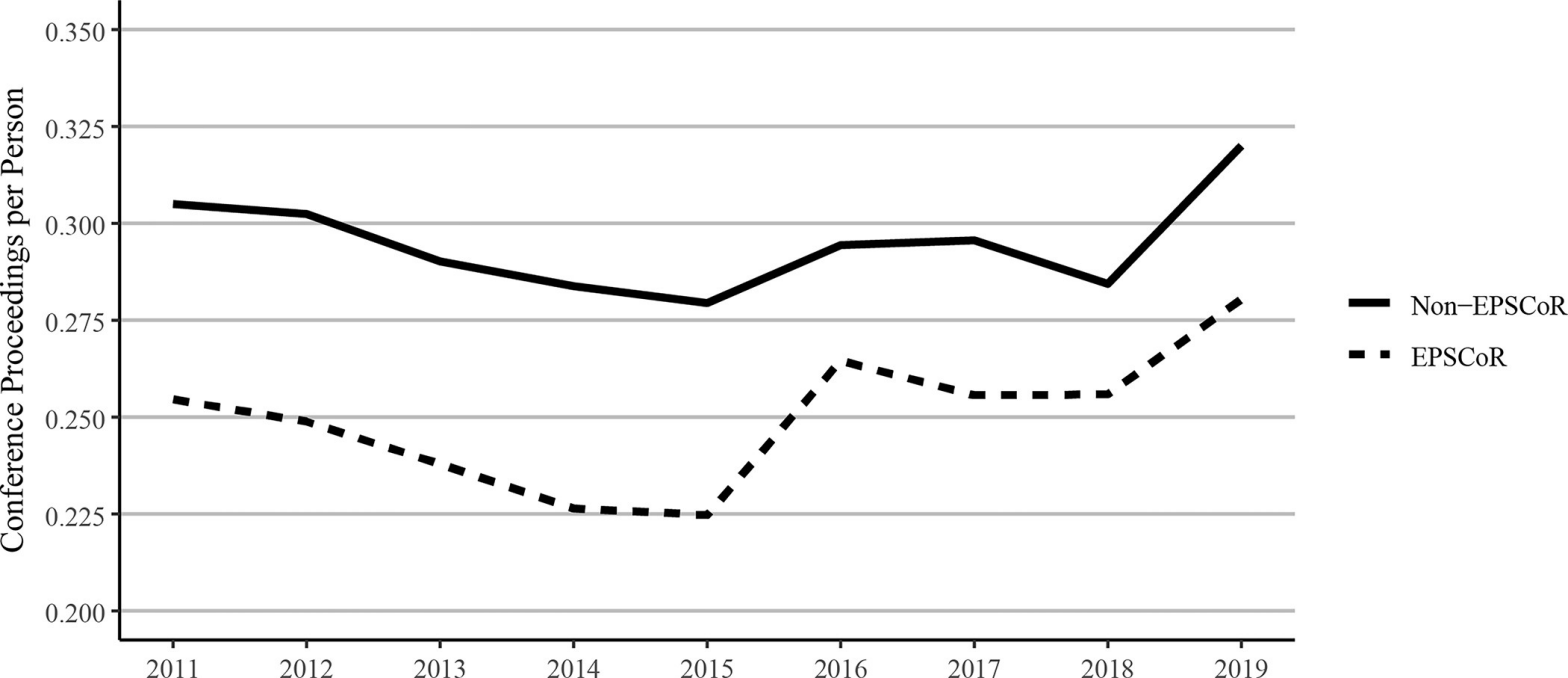
Conference proceedings per faculty member published between 2011 and 2019 at Ph.D. granting universities in EPSCoR and non-EPSCoR states (data for conference proceedings published in 2020 were not available at the time data were collected for this study). EPSCoR mean = 0.25 ±0.006; non-EPSCoR mean = 0.29 ±0.005; p < 0.01.

The number of conference proceedings publications, when expressed as per faculty member, was found to be less in EPSCoR vs non-EPSCoR states from 2011–2019 (Fig 6). During this period, the conference proceedings per person increased in both EPSCoR and non-EPSCoR states (Fig 6). Conference proceedings per person increased at a greater rate among EPSCoR states between 2011 and 2019 (10.1%) than in non-EPSCoR states (4.9%).

### 3.5. Citations per faculty member and citations per journal article

**Fig 7:**
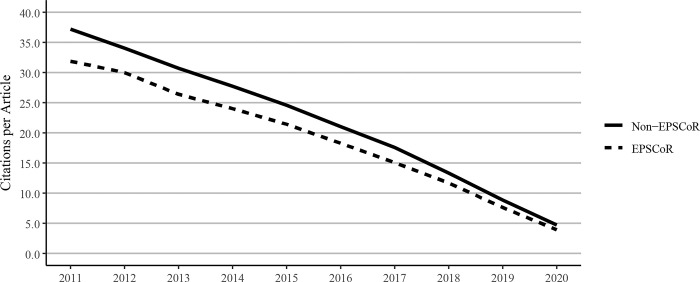
Citations to journal articles per faculty member at Ph.D. granting universities in EPSCoR and non-EPSCoR states between 2011 and 2020. Citation data were measured as of calendar year 2021; since older articles have had a greater time to collect citations, the chart shows a trend towards lower citations over time from the oldest to the newest articles. EPSCoR mean = 36.7 ±6.85; non-EPSCoR mean = 44.3 ±5.50; p < 0.01.

**Fig 8:**
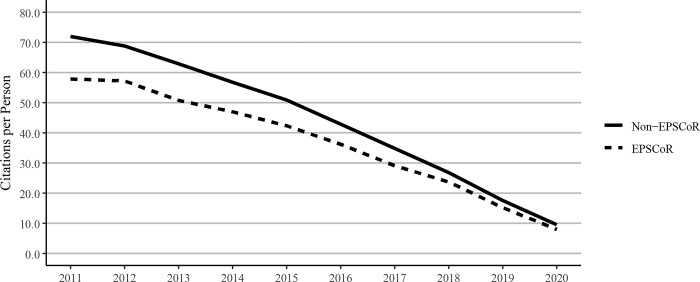
Citations per journal article at Ph.D. granting universities in EPSCoR and non-EPSCoR states between 2011 and 2018. Citation data were measured as of calendar year 2021; since older articles have had a greater time to collect citations, the chart shows a trend towards lower citations over time from the oldest to the newest articles. EPSCoR mean = 19.0 ±2.98; non-EPSCoR mean = 22.0 ±3.43; p < 0.01.

Citations to journal articles were tallied as of the calendar year 2021. Because older journal articles have a greater time to collect citations, our data showed a decreasing trend over time from the oldest to the newest articles. When citations of journal articles were expressed per faculty member (Fig 7) or as citations per journal article (Fig 8), non-EPSCoR states were found to have higher numbers of citations when compared to EPSCoR states.

### 3.6. Patents per faculty member

**Fig 9:**
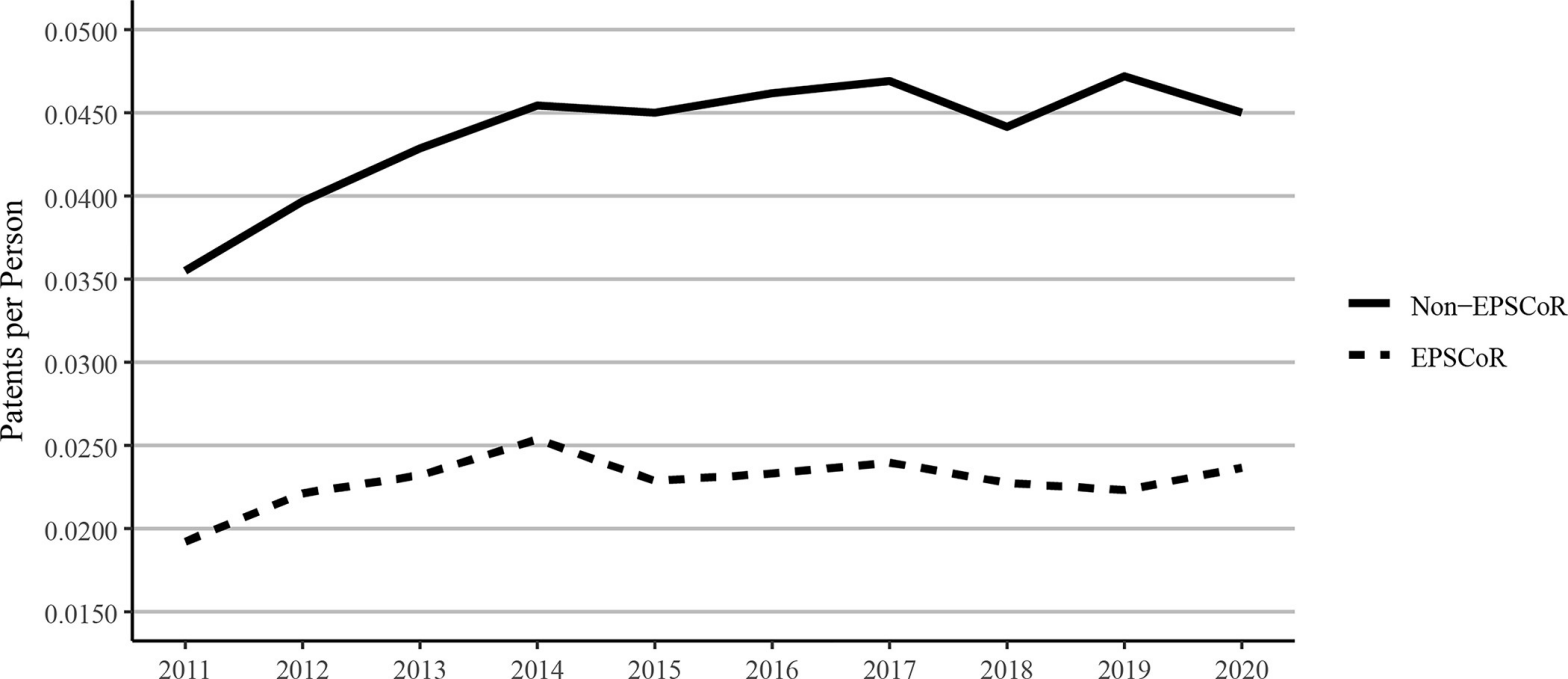
USPTO patents per faculty member published between 2011 and 2020 at Ph.D. granting universities in EPSCoR and non-EPSCoR states. EPSCoR mean = 0.023 ±0.0005; non-EPSCoR mean = 0.043 ±0.0011; p < 0.01.

Our data showed that 75,657 unique patents were granted by the USPTO to scholars in our sample between 2011 and 2020. The data on patents, when expressed on a per faculty basis, showed that the non-EPSCoR states performed better than EPSCoR states (Fig 9). Patent authorship increased at similar rates among EPSCoR faculty (23.1%) and non-EPSCoR faculty (26.8%) between 2011 and 2020 (Fig 9). Previous scholars quantified the increase in patent activity and patents granted to US universities [15, 16], and by some accounts, the number of patents granted to US universities doubled between 1996 and 2014 [17]. Our data are consistent with the reported increase in university patent activity and show little difference between EPSCoR and non-EPSCoR state universities in terms of the increase in patent production over time.

### 3.7. Scholarly outputs per million grant dollars

**Fig 10:**
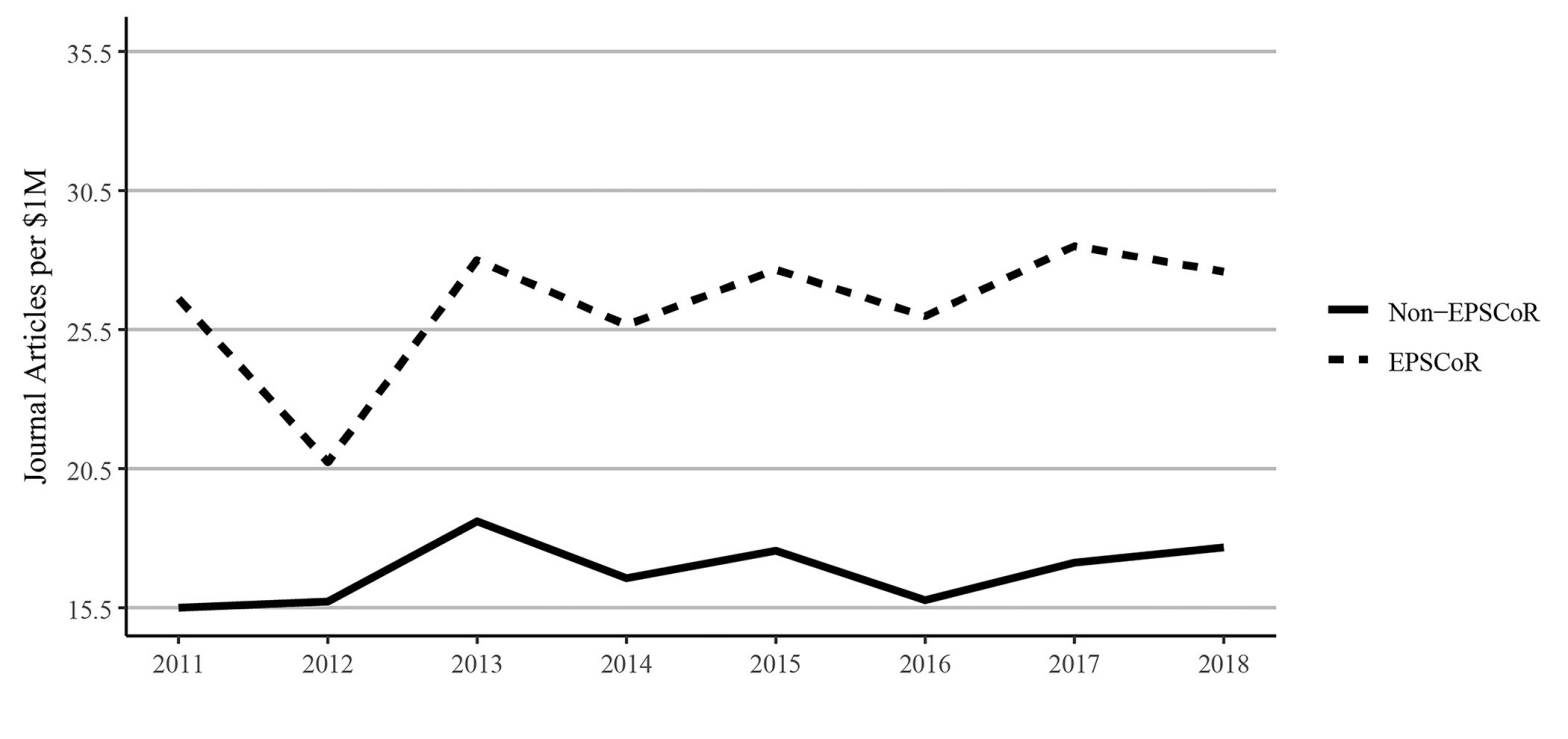
Journal article publications per $1 million federal research grant dollars at Ph.D. granting universities in EPSCoR and non-EPSCoR states between 2011 and 2018. EPSCoR mean = 27.9 ±1.27; non-EPSCoR mean = 18.3 ±1.12; p < 0.01.

**Fig 11:**
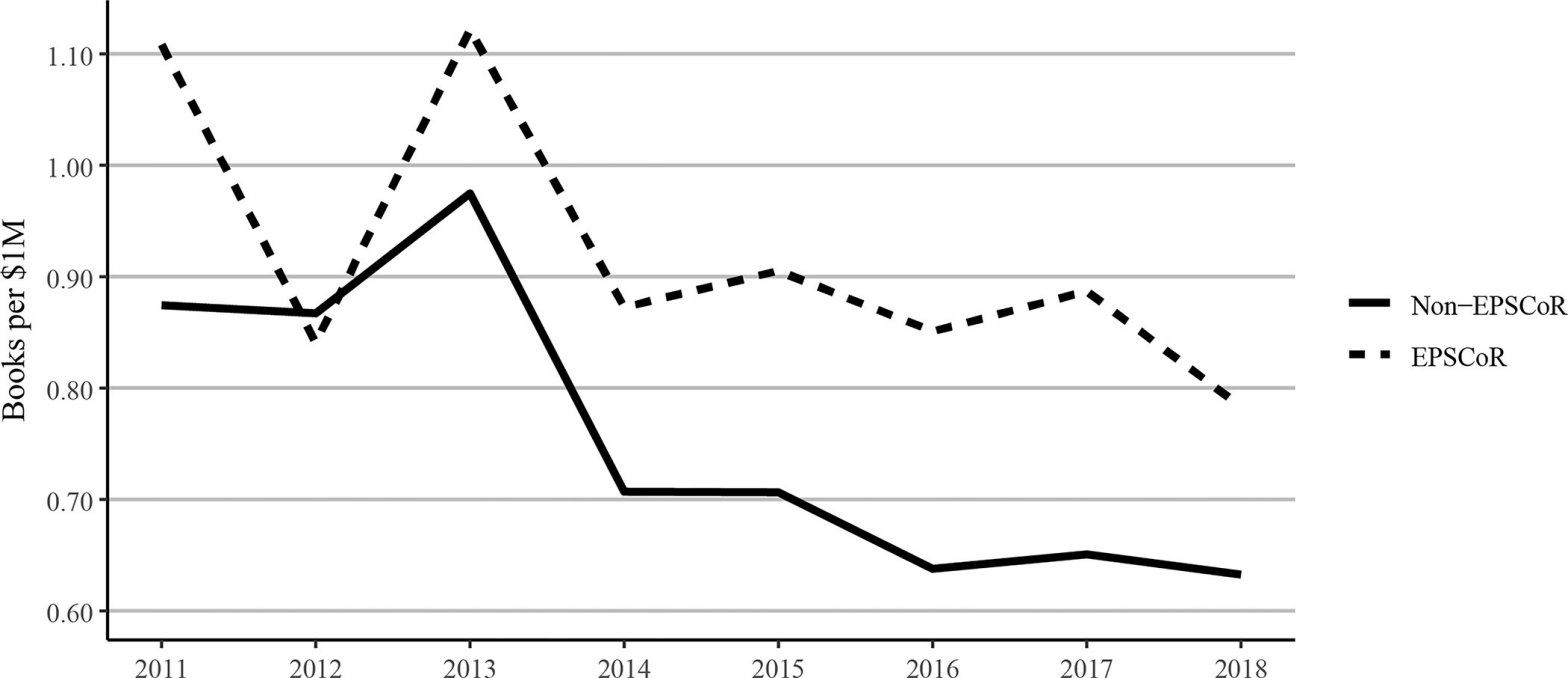
Book publications per $1million federal research grant dollars at Ph.D. granting universities in EPSCoR and non-EPSCoR states between 2011 and 2018. EPSCoR mean = 0.91 ±0.347; non-EPSCoR mean = 0.76 ±0.367; p < 0.01.

There is considerable discussion on whether the federal research funding invested in EPSCoR states yields results in promoting research productivity. In the current study, we, therefore, analyzed the data to understand the impact of every $1 million of federal investment on various research metrics described previously. Scaling scholarly outputs by federal grant dollars received, the data showed that EPSCoR states published substantially more journal articles per $1 million in federal funding received between 2011 and 2018 (Fig 10). Book publications per $1 million of federal research funding showed declines in both EPSCoR and non-EPSCoR states (Fig 11), consistent with our finding that book publications overall had declined over this period (Fig 5).

**Fig 12:**
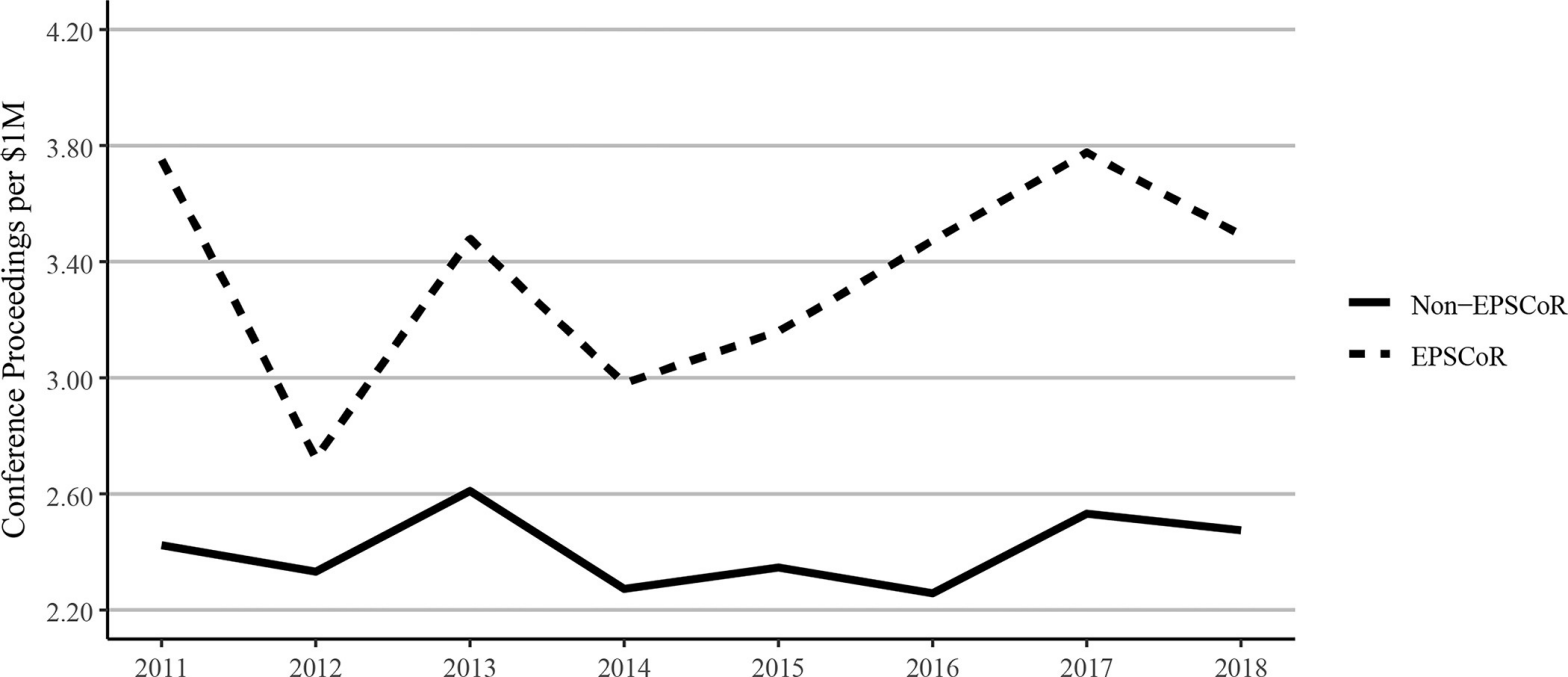
Conference proceedings publications per $1million federal research grant dollars at Ph.D. grating universities in EPSCoR and non-EPSCoR states between 2011 and 2018. EPSCoR mean = 3.5 ±0.17; non-EPSCoR mean = 2.6 ±0.14; p < 0.01.

**Fig 13:**
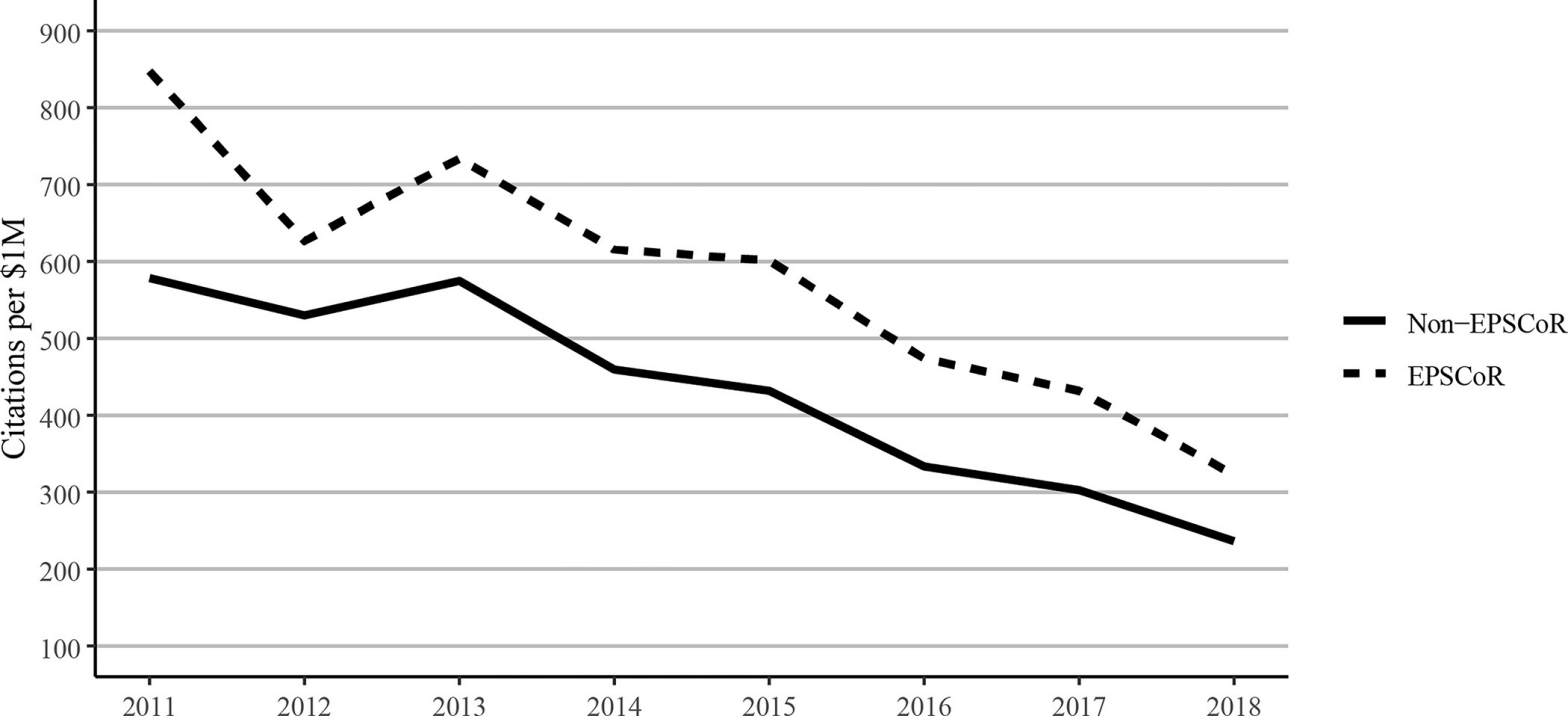
Citations to journal articles per $1 million federal research grant dollars at Ph.D. granting universities in EPSCoR and non-EPSCoR states between 2011 and 2018. Citation data were measured as of calendar year 2021; since older articles have had a greater time to collect citations, the chart shows a trend towards lower citations over time from the oldest to the newest articles. EPSCoR mean = 504 ±70.3; non-EPSCoR mean = 376 ±51.4; p < 0.01.

**Fig 14:**
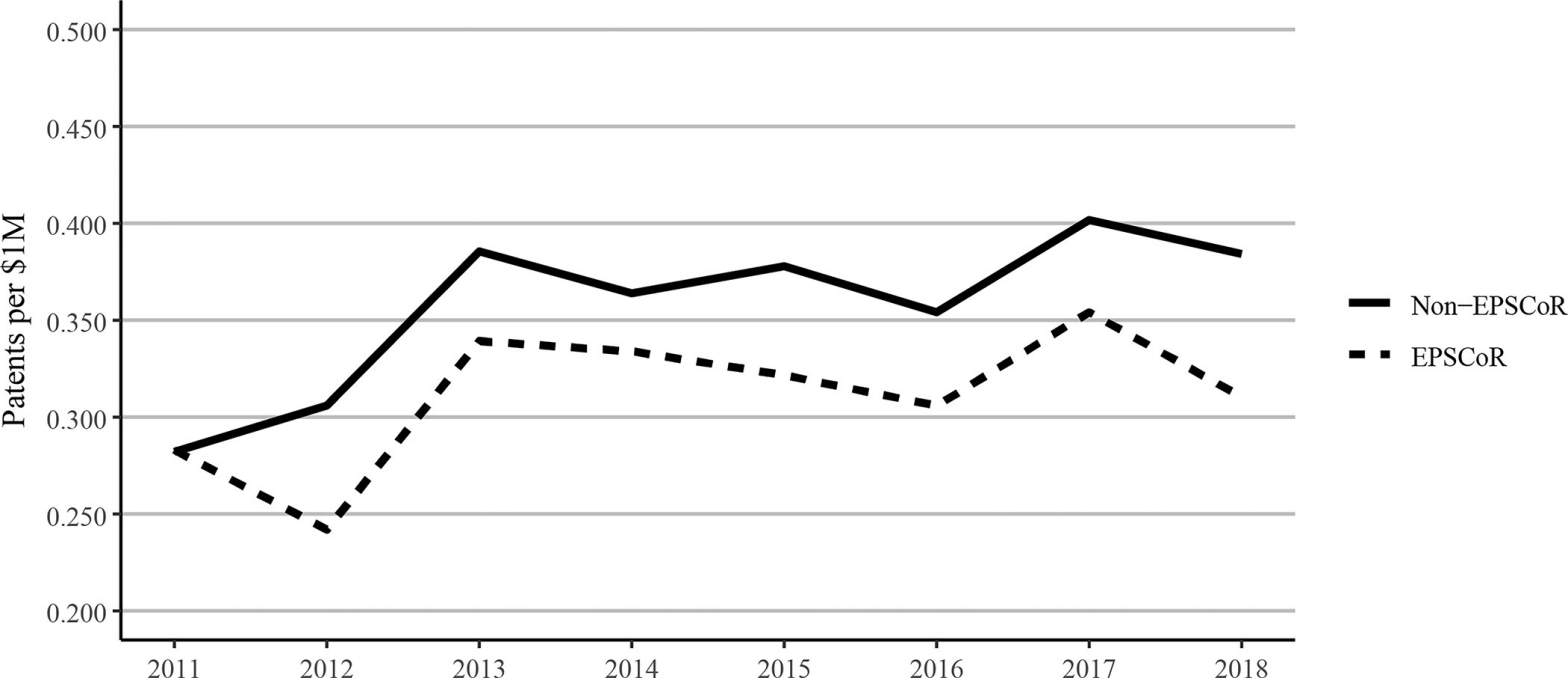
Patents per $1 million federal research grant dollars at Ph.D. granting universities in EPSCoR and non-EPSCoR states between 2011 and 2018. EPSCoR mean = 0.33 ±0.015; non-EPSCoR mean = 0.39 ±0.029; p < 0.01.

However, EPSCoR states showed greater book publications when compared to non-EPSCoR states per $1 million in federal grants. Conference proceedings per $1 million in federal research funding follow a similar pattern as journal articles, with EPSCoR states producing a greater number of proceeding publications than non-EPSCoR states (Fig 12). The citations per $1M federal research funding showed that EPSCoR states had higher numbers of citations vs non-EPSCoR states (Fig 13). Finally, patents were the only category in which the non-EPSCoR states performed better than EPSCoR states per $1M federal research funding over the study period (Fig 14).

## 4. Discussion and study limitations

In the current study, we investigated the combined research output of Ph.D. granting universities in EPSCoR and non- EPSCoR states and found that the non-EPSCoR received ∼5 times more federal research funding when compared to the EPSCoR states. One of the clear reasons for this disparity was that in non-EPSCoR states, there was a greater number of Ph.D. granting universities (316) when compared to the EPSCoR states (76). This also resulted in significantly more numbers of faculty in non-EPSCoR vs EPSCoR states. While the size of the faculty accounts for the disparity seen in federal dollars received by non-EPSCoR vs EPSCoR states, there could be additional factors such as lack of advanced research infrastructure and support as well as fewer numbers of Carnegie R1 universities in EPSCoR states when compared to the non-EPSCoR states. An NSF-sponsored 2M report on EPSCoR published in 2022 also suggested several differences between the EPSCoR vs non-EPSCoR states (https://nsf-gov-resources.nsf.gov/2022-06/EPSCoR%20Base%20Period%20Final%20Report%20-%20%28508%20Compliant%29.pdf). This report noted that most EPSCoR states had a smaller economic base, awarded a smaller number of Science and Engineering degrees, and received less federal funding possibly because of lower numbers of doctoral granting universities when compared to the non-EPSCoR states. Also, the EPSCoR state governments were found to provide less support for R&D activities when compared to the non-EPSCoR states.

Because of the significant differences in the total numbers of faculty in EPSCoR vs non-EPSCoR states, we expressed the data on per capita basis. The data on journal article publications showed that while non-EPSCoR faculty members performed better than EPSCoR counterparts initially (2011–2017), this gap narrowed from 2018–2020 (Fig 4). However, with book chapters, conference proceedings, and especially with the patents, the non-EPSCoR states performed better than the EPSCoR states. The increase in research productivity seen with non-EPSCoR faculty can be explained by the fact that per capita, the non-EPSCoR faculty received more research funding than their EPSCoR counterparts. The larger size of grants may help support more graduate students and post-doctoral scholars and, thus, more research productivity.

While larger grants do not necessarily translate into more research productivity, we tested this notion by analyzing and expressing the data on research productivity per $1 million in federal research funding. We felt that such data also address the question of whether additional investment by NSF and other federal agencies in EPSCoR programs, as proposed in the recently passed CHIPS and Science act will yield positive results. Interestingly, such analysis showed that for every $1 million in federal investment in research, the EPSCoR states performed significantly better than non-EPSCoR states in virtually all categories, including journal articles, book chapters, and conference proceedings, and citations, except patents (Figs 10–14). Journal article publications are considered the prime indicators of the success of a research program, and publishing articles is an expectation of federal grant support. Thus, while the data expressed in journal articles on per capita basis showed that non-EPSCoR states were performing better, the gap was narrowing in more recent years (2018–2020). Also, when the data on journal articles was expressed as per $1 million in research funding, the EPSCoR states performed better than non-EPSCoR states.

Overall, the data suggested that while non-EPSCoR states received significantly higher levels of federal research funding from 2011–2020 when compared to the EPSCoR states, the per capita research productivity is only modestly higher. Moreover, for every dollar of federal investment in research, the EPSCoR states performed better than non-EPSCoR states in overall research productivity. This can be explained by the findings that while the non-EPSCoR states received ~5 times more federal funding, their research output in various indicators tested was not proportional to this increased level of funding.

We view these results as an early indication of the success of the EPSCoR states despite the limitations of lacking robust research infrastructure, we caution that the present study should be expanded to control for other variables. Specifically, future versions of this study will include the following controls:

1. Each academic discipline has a unique culture of scholarly dissemination [18, 19]. While journal article publications are the most common form of scholarly research dissemination in most disciplines, humanities scholars publish more books than articles, and some engineering fields publish more conference proceedings than journal articles. Our comparisons of EPSCoR and non-EPSCoR states would benefit from controlling, state-by-state, for the population of scholars in each discipline, so publication cultures can be normalized and accounted for. Likewise, some disciplines necessitate greater research funding than others (*e*.*g*., laboratory sciences compared to language and literature disciplines), and the distribution of disciplines across states may be used to normalize funding expectations and requirements.
2. An additional limitation of this study with respect to the journal articles is that not all articles counted were supported by federal funding for research. However, such a limitation applies to both EPSCoR and non-EPSCoR states. Thus, additional studies are necessary to sort out those publications that specifically acknowledge federal grant support.
3. Research quantity is not a measure of research quality. While EPSCoR and non-EPSCoR states show different patterns of journal article publications when scaled by faculty count or federal research investment, those trends should be contextualized by exploring the impact of those works. The citation data are one of the indicators of research impact. However, other impacts of research include innovation, job creation, and economic growth.
4. Although relatively rare and lower in dollar value than federal research grants, the availability of state-sponsored research grants may also be considered as a means to control each state’s investment in higher education research funding.
5. Teaching loads are likely to vary between institutions (*e*.*g*., Carnegie “R1” vs “R2” institutions). Our study would benefit from considering the relative number of institutions and faculty represented by each institutional type to understand whether some states are characterized by greater average teaching obligations than others and whether this important function of those universities is related to the quantity of their research outputs.
6. Likewise, the number of doctoral students and post-docs whose employment is funded by federal grant dollars, as well as the number of research dollars dedicated to infrastructure, may be greater at “R1” institutions than “R2” institutions. Thus, the dollars available for research costs *sensu stricto* (as opposed to salary and infrastructure) may not be reflected in the total grant dollars as measured in this study. As above, controlling for institution type may allow us to better understand the total dollars available for direct research costs.
7. The EPSCoR and non-EPSCoR groups both show considerable within-group variability. Beyond reporting averages by group, this analysis would benefit from a discussion of within-group variability and an exploration of outlier states which produce a much larger proportion of scholarly works per person and per dollar than expected. Outlier analysis, perhaps combined with data transformations (*e*.*g*., to a log scale) may reveal new patterns and facilitate more nuanced comparisons between groups. Data transformations and state-level analysis will also be necessary before performing more sophisticated regression analyses seeking to understand the relationships between the variables we employed.
8. Our analysis was limited to only Ph.D. granting institutions. In addition to excluding Ph.D. granting universities in Puerto Rico and the US Virgin Islands due to the unavailability of data in the AcA database for these institutions, this limitation also excludes other types of institutions where federally funded research may be an important part of the research enterprise (*e*.*g*., liberal arts universities, masters-focused universities). Including a wider range of institutional types may offer additional insights into the impact of federal research dollars on EPSCoR states. While our studies compared the overall impact of federal research funding on EPSCoR vs non-EPSCOR states, the impact of specific EPSCoR funding on the EPSCoR jurisdictions was not studied. In this regard, a 2011 NSF- sponsored report from the IDA Science and Technology Policy Institute (STPI) indicated that EPSCoR did play a critical role in increasing NSF funding in early stages but not at later time points (https://www.ida.org/-/media/feature/publications/e/ev/evaluation-of-the-national-science-foundations-experimental-program-to-stimulate-competitive-researc/p-5221.ashx). However, this report found that the EPSCoR programs were helping the universities recruit new faculty, expand their research bases, and enhance Science and Engineering research and education programs.

## 5. Conclusion

Our analysis revealed that non-EPSCoR states received more research funding per faculty and exhibited more research productivity when compared to their EPSCoR counterparts. Interestingly, however, for every $1 million of federal investment in research, EPSCoR states exhibited overall, more research productivity than the non-EPSCoR states. Promoting geographic diversity in federal research funding is critical to ensure research competitiveness and economic growth across all states in the U.S. However, whether the states currently receiving significantly less research funding are competitive enough to make the best use of additional resources if allocated is unclear. Our studies on research productivity in EPSCoR vs non-EPSCoR states reveal that EPSCoR states are performing very well and are well poised to grow their research capacity. Thus, strategic research investments in such states could contribute towards the overall research and economic competitiveness of the U.S.
